# The impact of sulfate restriction on seed yield and quality of winter oilseed rape depends on the ability to remobilize sulfate from vegetative tissues to reproductive organs

**DOI:** 10.3389/fpls.2014.00695

**Published:** 2014-12-17

**Authors:** Alexandra Girondé, Lucie Dubousset, Jacques Trouverie, Philippe Etienne, Jean-Christophe Avice

**Affiliations:** ^1^Normandie UniversityCaen, France; ^2^Université de Caen Basse Normandie, UMR INRA–UCBN 950 Ecophysiologie Végétale, Agronomie and Nutritions N.C.S.Caen, France; ^3^Institut National de la Recherche Agronomique, UMR INRA–UCBN 950 Ecophysiologie Végétale, Agronomie and Nutritions N.C.S.Caen, France

**Keywords:** *Brassica napus* L., sulfate restriction, ^34^S labeling, S flux, sulfate transporter, seed yield, seed quality

## Abstract

Our current knowledge about sulfur (S) management by winter oilseed rape to satisfy the S demand of developing seeds is still scarce, particularly in relation to S restriction. Our goals were to determine the physiological processes related to S use efficiency that led to maintain the seed yield and quality when S limitation occurred at the bolting or early flowering stages. To address these questions, a pulse-chase ^34^SO^2−^_4_ labeling method was carried out in order to study the S fluxes from uptake and remobilization at the whole plant level. In response of S limitation at the bolting or early flowering stages, the leaves are the most important source organ for S remobilization during reproductive stages. By combining ^34^S-tracer with biochemical fractionation in order to separate sulfate from other S-compounds, it appeared that sulfate was the main form of S remobilized in leaves at reproductive stages and that tonoplastic SULTR4-type transporters were specifically involved in the sulfate remobilisation in case of low S availability. In response to S limitation at the bolting stage, the seed yield and quality were dramatically reduced compared to control plants. These data suggest that the increase of both S remobilization from source leaves and the root proliferation in order to maximize sulfate uptake capacities, were not sufficient to maintain the seed yield and quality. When S limitation occurred at the early flowering stage, oilseed rape can optimize the mobilization of sulfate reserves from vegetative organs (leaves and stem) to satisfy the demand of seeds and maintain the seed yield and quality. Our study also revealed that the stem may act as a transient storage organ for remobilized S coming from source leaves before its utilization by seeds. The physiological traits (S remobilization, root proliferation, transient S storage in stem) observed under S limitation could be used in breeding programs to select oilseed rape genotypes with high S use efficiency.

## Introduction

Oilseed rape (*Brassica napus* L.) is a crop that produces seeds with high contents of oil and proteins for human and animal nutrition as well as non-edible uses. To provide an adequate amount of S for oilseed rape culture, the recommendations are about 20–30 kg S.ha^−1^. Oil and protein concentrations in seeds have been shown to increase with S fertilization (Ahmad et al., 2007; Malhi et al., 2007). In addition, the application of S fertilizers also improves N-use efficiency and maintains a sufficient oil level and fatty acid quality in oilseed rape (Schnug et al., 1993; Fismes et al., 2000; Dubousset et al., 2010). In modern-grown oilseed rape varieties (i.e., double low varieties with zero erucic acid and a reduced content of seed glucosinolates), the S harvest index (S content in seeds divided by the total S in the whole crop) is typically only 20% (McGrath and Zhao, 1996). This indicates that a large proportion of S taken up by the crop is retained in the vegetative tissues and pods.

In order to increase crop competitiveness, the oil yield needs to be improved while limiting fertilizer inputs. Compared with other crops such as cereals, oilseed rape is particularly sensitive to S deficiency because it has a high demand for S (Zhao et al., 1997). The reduction in atmospheric deposition of S has increased the incidence of S deficiency in oilseed rape (McGrath and Zhao, 1996). S deprivation in oilseed rape leads to reduced seed yields and oil quality (Janzen and Bettany, 1984; Scherer, 2001). Recent work based on proteomics approaches in mature seeds obtained from winter oilseed rape plants grown under low sulfate applied at the bolting, early flowering or start of pod filling stages (D'Hooghe et al., 2014) have revealed that the protein quality of seeds was reduced depending on the severity of S limitation and was associated with a reduction in S-rich seed storage protein accumulation (such as Cruciferin Cru4) which favored S-poor seed storage protein (such as Cruciferin BnC1). Nevertheless, our knowledge about the stages of development the more sensitive to S limitation or the physiological processes that are involved in S management by oilseed rape subjected to sulfate restriction remains largely unclear (Dubousset et al., 2010).

In oilseed rape, S is mainly taken up by the roots as sulfate (SO^2−^_4_) and transported *via* the xylem to the leaves by specific transporters (Hawkesford and De Kok, 2006; Takahashi et al., 2011). Sulfate is mainly reduced to Cysteine (Cys) in leaves, and either converted to Methionine or incorporated into proteins, Cys-containing peptides such as glutathione, or numerous secondary metabolites involved in plant defense (Sulfur-containing Defense Compounds, SDC) such as glucosinolates. The root uptake and subsequent distribution of SO^2−^_4_ to the leaves is closely related to growth demand and mineral S availability in the soil. Sulfate stored in vacuoles is the main form of S released by the mesophyll cells under low S nutrition conditions (Blake-Kalff et al., 1998; Scherer, 2001; Matula and Pechová, 2002; Parmar et al., 2007). Indeed, a reduction in the sulfate supply leads to an up-regulation of genes encoding for specific transporters involved in (i) sulfate uptake by roots, and (ii) sulfate remobilization at the level of roots and leaves, especially *BnSultr4;1* and *BnSultr4;2*, which are involved in vacuolar efflux of sulfate (Parmar et al., 2007; Dubousset et al., 2009; Abdallah et al., 2010).

The leaves play a crucial role in recycling foliar compounds to sustain seed filling during the reproductive stages and therefore contribute to the maintenance of seed yield of oilseed rape. Noquet et al. (2004) have reported that ablation of 50% of the leaves present at the bolting stage results in a 30% decrease in seed yield in oilseed rape. In the case of a transient mineral S limitation occurring at the rosette stage, winter oilseed rape is able to maintain its growth by optimization of the recycling of endogenous foliar S compounds (particularly sulfate) from old and mature leaves without any acceleration of leaf senescence processes (Dubousset et al., 2009; D'Hooghe et al., 2013). Although mobilization of S from vegetative tissues is likely to be crucial for seed filling, very little is known about the dynamics, the efficiency and the contribution of S mobilization from vegetative tissues to seeds in oilseed rape.

In this study we investigated the S management of winter oilseed rape in response to S restriction applied at two crucial growth stages (bolting or early flowering). The impact of sulfate limitation was studied on seed yield and quality. To evaluate the level of S remobilization from vegetative parts using stable isotope as tracer, a greenhouse experiment was carried out for long-term steady state ^34^S-SO^2−^_4_ pulse labeling. This method is particularly appropriate for studying S management at the whole plant level and for showing the source/sink relationships for S from uptake or remobilisation. In addition, fluxes of S remobilization were studied in relation to the dynamics of mobilization of S compounds (sulfate vs. S-reduced compounds) as well as the gene expression of sulfate transporters (SULTR) of group 4 in response to sulfate availability in the main source organs i.e., mature leaves.

## Materials and methods

### Experimental treatments, mineral S restriction and ^34^S labeling

The experimental design was previously described by Dubousset et al. (2010). Briefly, seedling of winter oilseed rape (*Brassica napus* cv Capitol) were first grown for 36 days on vermiculite under a thermoperiod of 20°C (day-16 h) and 15°C (night-8 h). To initiate flowering and the reproductive stages of development, a period of vernalization was applied to plants for 46 days, consisting of a thermoperiod of 10°C (day 10 h) and 4°C (night 14 h). During pre-culture and vernalization, light was supplied by High Pressure Sodium Lamps (Philips, MASTER GreenPower T400W) with a PAR (Photosynthetically Active Radiations) of 400 μmol photon.s^−1^.m^−2^ at the top of the canopy, and plants were supplied with 25% Hoagland nutrient solution (1.25 mM Ca(NO_3_)_2_,4H_2_O, 1.25 mM KNO_3_, 0.5 mM MgSO_4_, 0.25 mM KH_2_PO_4_, 0.2 mM EDTA 2NaFe,3H_2_O, 14 μM H_3_BO_3_, 5 μM MnSO_4_, 3 μM ZnSO_4_, 0.7 μM (NH_4_)_6_Mo_7_O_24_, 0.7 μM CuSO_4_, 0.1 CoCl_2_) renewed twice a week.

After vernalization, plants were individually transferred into 2.5 L pots containing mixed vermiculite (1V) and perlite (2V). Greenhouse conditions were maintained at a thermoperiod of 20°C (day-16 h) and 15°C (night-8 h). Plants grew under natural light conditions and received 25% Hoagland nutrient solution as described above. In order to determine the endogenous and exogenous S fluxes at the whole plant level, a long term pulse-chase ^34^S labeling (^34^SO^2−^_4_ at 1 atom% excess) was applied from the end of the vernalization period up to the beginning of the S limitation treatments. S restriction (Low S: LS) equivalent to 8.7 μM sulfate was applied at two different stages of development: at GS32 (bolting stage, LS32 plants) or GS53 (early flowering, LS53 plants). When S restriction started, ^34^S-labeling was stopped and unlabeled-sulfate was supplied in the nutrient solution until the final stage of development (GS99: mature seeds). For each S limitation treatment, control plants (High S treatment: HS) supplied with 508,7 μM sulfate were also submitted to the same period of pulse-chase ^34^S labeling in order to compare the S fluxes between S-limited and non-limited (control) plants. LS and HS plants were harvested at GS32, GS53, GS70 (first pods), GS81 (seed coloring) and GS99 (mature seeds). Plants were separated into roots, leaves, stems, floral stems, pod walls and seeds. The age of leaves were determined by the relative chlorophyll concentration using a non-destructive SPAD (Soil Plant Analysis Development) chlorophyll meter (Minolta, SPAD-502 model) and the measurement of leaf area using a LI-COR 300 area meter (LI-COR, Lincoln, NE, USA). Based on leaf area and chlorophyll level, two mature leaves (leaf ranks 7 and 8) that became source for S during the experiment, were chosen for further biochemical and molecular analyses (sulfate, residual S in dead leaves, expression of sulfate transporters of group 4) in relation to S remobilization. Each organ was freeze-dried, weighed and ground to a fine powder to be analyzed. Aliquots of fresh matter were also stored at −80°C until further gene expression analyses.

### Determination of total S amount, ^34^S amount and S fluxes

For each organ and each date of harvest, S and ^34^S analyses were performed with an elemental analyser (EA3000, EuroVector, Milan, Italy) linked to a continuous flow isotope mass spectrometer (IRMS, IsoPrime GV instruments, Manchester, UK). The long-term period of isotope labeling described previously allowed a homogenous incorporation of tracers into the whole plant. Therefore, the amount of ^34^S in excess was proportional to the amount of S and allowed identification of the “sink” or “source” status of organs. As such, an organ was considered as “a sink” if a gain in the ^34^S amount was observed for a period Δ*t*. Inversely, a “source” organ was characterized by a loss in the amount of ^34^S for a period Δ*t*. For the determination of S fluxes (Salon et al., 2014), it is necessary to quantify the ^34^S amount in excess (Q^34^S_in excess_) in each organ. For this, the value of isotope abundance (A%) given by the IRMS was used as follows:

$${\text{Q}}^{34}{\text{S}}_{\text{in excess}}(\text{mg})=[(\text{A}{\%}_{\text{sample}}-\text{A}{\%}_{\text{natural standard}})/100]\times \text{QS}$$

where A%_natural standard_ corresponds to natural ^34^S abundance i.e., 4.2549%, QS is the amount of total S in a given organ in mg, and A%_sample_ corresponds to ^34^S isotope abundance in a given organ. A% in a sample or natural standard is calculated as:

$$\text{A}\%=100\times \;\left[{}^{34}\text{S}/\left({}^{34}\text{S}{+}^{\text{32S}}\right)\right]$$

For every organ on each date, ^34^S amounts were normalized (Q^34^S _normalized_) with the following calculation at every date (*t*):

$$\begin{array}{l} {\text{Q}}^{34}{\text{S}}_{\text{normalized}}=({\text{Q}}^{34}\text{S}t_{\text{organ}}\times \text{Average }{\text{Q}}^{34}\text{S}\\ \;\text{whole }{\text{plant}}_{\left(\text{all dates included}\right)})/{\text{Q}}^{34}\text{S}t_{\text{whole plant}} \end{array}$$

where Q^34^S*t*_organ_ indicates the ^34^S amount in excess in a given organ at the date *t*, Average Q^34^S whole plant _(all dates included)_ represents the average ^34^S amount in the whole plant at every study date and Q^34^S*t*_whole plant_ indicates the ^34^S amount in the whole plant at the date *t*.

For source organs (decline in the ^34^S amount between *t* and *t* + Δ*t*), the S amount remobilized (QSR_source_) was defined as:

$${\text{QSR}}_{\text{source}}=\left[{\text{QS}}_{\text{total}}t\times \left({\text{Q}}^{34}\text{S}t-{\text{Q}}^{34}\text{S}t+\Delta t\right)\right]/{\text{Q}}^{34}\text{S}t$$

where QS_total_*t* indicates total S amount in the source organ at the date *t*, Q^34^S*t* represents the ^34^S amount in excess in the source organs at the date *t* and Q^34^S*t* + Δ*t* indicates the ^34^S amount in excess in the source organs at *t* + Δ*t*.

For sink organs (gain of ^34^S amount between *t* and *t* + Δ*t*), the S amount remobilized (QSR_sink_) corresponded to:

$$\begin{array}{l} {\text{QSR}}_{\text{sink}}=\left({\text{Q}}^{34}\text{S}t+\Delta t-{\text{Q}}^{34}\text{S}t\right)/[\left(\Sigma {\text{Q}}^{34}{\text{S}}_{\text{source organs}}t\right)\\ \;-\left(\Sigma {\text{Q}}^{34}{\text{S}}_{\text{source organs}}t+\Delta t\right)/{\text{QS}}_{\text{total}}\text{ remobilized}] \end{array}$$

where Σ Q^34^S_source organs_*t* indicates the total ^34^S amount in excess from source organs at *t*, Σ Q^34^S_source organs_ *t* + Δ*t* represents the total ^34^S amount in excess from source organs at *t* + Δ*t* and QS_total_ remobilized indicates the total S amount in excess remobilized (from all source organs).

The inflow of S taken up was also calculated between *t* and *t* + Δ*t*.

For source organs, the S amount taken up (QSI_source_) was defined as:

$${\text{QSI}}_{\text{source}}=(\text{QS}t+\Delta t-\text{QS}t)+{\text{QSR}}_{\text{source}}$$

For sink organs, the S amount taken up (QSI_sink_) corresponded to:

$${\text{QSI}}_{\text{sink}}=(\text{QS}t+\Delta t-\text{QS}t)-{\text{QSR}}_{\text{sink}}$$

where QSR_source_ indicates the S amount from the studied source organ and QSR_sink_ indicates the S amount from the studied sink organ.

### Sulfate amount and determination of ^34^S in sulfate

Forty-five milligrams of lyophilized powder from source leaves were used to extract sulfate after two successive incubations with 2 mL of 50% ethanol at 45°C for 1 h, centrifugation at 10,000 g for 20 min and two incubations with water at 95°C for 1 h, ending with centrifugation at 10,000 g for 20 min. The supernatants were pooled and evaporated under vacuum (Concentrator Evaporator RC 10.22, Jouan, Saint-Herblain, France). The dry residue was re-suspended in 2 mL of ultra-pure water and the sulfate concentration was determined by high performance liquid chromatography (HPLC; DX100, DIONEX Corp, Sunnyvale, CA, USA). This extract contains sulfate and other soluble S compounds. Therefore, in order to determine the contribution of sulfate to the S remobilization from source leaves it was necessary to purify sulfate and quantify the changes in S-sulfate and ^34^S-sulfate. Thus, this extract passed through a DOWEX 50W (H+) column (4 cm long, 1 cm of diameter) and was eluted with 7.5 mL of water. The resulting eluted fraction contained purified sulfate. A part of this fraction was evaporated under vacuum and finally re-suspended with ultra-pure water in a volume to obtain an S concentration of around 0.85 μg S.μL^−1^. Thirty microliters were placed in a tin capsule and the S and ^34^S amounts were determined with an elemental analyser coupled with IRMS as described above. The rest of the fraction (about 1.5 mL) was used to determine the sulfate concentration by HPLC (as described above) in order to verify the purity of the sulfate fraction after comparison with the IRMS data.

### Determination of oil and glucosinolate contents by NIRS

The seed samples were scanned on a monochromator NIR system model 6500 (FOSS NIRSystems Inc, Silver Spring, MD, USA) equipped with the transport module, in the reflectance mode. Intact seeds (about 5 g) were placed in a standard ring cup and scanned. The results were predicted from an external calibration established for oil and total glucosinolate content (CRAW, Gembloux, Belgium). Three determinations were performed for each sample. The results were given in % of oil per DM seed and in μmol of total glucosinolates per g of DM seed.

### Determination of oil content and lipid composition of seeds

The method for determination of seed oil content was based on direct methylation of fatty acids. Briefly, 10 mature dried seeds were ground in a microtube tube containing 3 inox balls using a Tissue Lyser system (Qiagen, Chatsworth, CA, USA). For each sample, three aliquots of 10 mg were weighed and transferred into glass tubes with Teflon-lined screw caps containing 1.32 mL of a freshly prepared solution of methanol/sulfuric acid/toluene (100/2.5/30, v/v/v) with 400 μg.mL^−1^ of heptadecanoic acid as an internal standard. The mixture was shaken vigorously for 30 s and then heated at 95°C for 1 h. After cooling on ice for 10–15 min, fatty acid methyl esters (FAMEs) were extracted into 500 μL of hexane following the addition of 1 mL of water. After vigorous hand shaking (15 s) and centrifugation (5 min, 2000 rpm), 10 μL of the upper organic phase was analyzed by gas chromatography (GC). If necessary, extracts were evaporated under nitrogen and dissolved into 50 μL of hexane before GC analysis. FAMEs were separated on a DB-WAX column (30 m l. × 0.25 mm i.d., 25 μm film, J&W Scientific Columns from Agilent Technologies Co., Palo Alto, CA, USA) and quantified with a flame ionization detector. The GC conditions were as follows: split mode injection (1:100), injector temperature at 250°C, oven temperature fixed at 220°C for 15 min and detector temperature at 260°C, with hydrogen as the carrier gas at a pressure of 100 kPa and a flow rate of 1 mL.min^−1^, in a 6890N GC GLC system (Agilent Technologies). To determine the mass of each fatty acid, the peak area was compared to the internal standard peak area. Results were given in μg of fatty acid per mg of DM seed and were expressed as % of control (HS plants).

### Transcriptomic analysis

Total RNA was extracted from 200 mg of fresh matter previously ground to a powder in a mortar containing liquid nitrogen and immersed in a hot mixture (80°C) containing 750 μL of phenol pH 4.3 and 750 μL of extraction buffer (0.1 M LiCl, 0.1 M Tris Base, 10 mM EDTA, 1% SDS (w/v), pH 8). Samples were vortexed for 40 s and 750 μL of chloroform/isoamylalcohol (24/1 v/v) were added. Samples were vortexed for 40 s again and centrifuged at 1500 g (4°C, 5 min). The supernatant was transferred to 750 μL of LiCl (4M) and incubated overnight at 4°C. After incubation, samples were centrifuged at 1500 g (4°C, 20 min). The supernatant was discarded and the pellet was suspended in 100 μL of sterile water. Then, RNA purification was made by Qiagen® RNeasy Kit according to the manufacturer's recommendation. RNA quantity was measured by a spectrophotometer (BioPhotometer, Eppendorf®) at 260 nm and their quality was check on an agarose gel (1.2% (w/v)).

A reverse transcription (RT) was performed on the purified RNA. For this, 1 μg of RNA was converted to cDNA using an “iScript cDNA synthesis kit” (Bio-Rad, Marne-La-Coquette, France) according to the manufacturer's recommendation. The obtained cDNA was used to perform Q-PCR using specific primers for *Brassica napus BnSultr4;1* (Accession no: AJ416461; forward primer: 5′-GACCAGACCCGTTAAGGTCA-3′, reverse primer: 5′TTGGAATCCATGTGGAAGCAA-3′) and *BnSultr4;2* (Accession no: FJ688133; forward primer: 5′-AGCAAGATCAGGGATTGTGG-3′, reverse primer: 5′-TGCAACATTTGTGGGTGTCT-3′) (Dubousset et al., 2009). Two internal control genes were used in this experiment: *EF1*-α (Accession no: DQ312264; forward primer: 5′-GCCTGGTATGGTTGTGACCT-3′, reverse primer: 5′-GAAGTTAGCAGCACCCTTGG-3′) and *18S RNA* (Accession no: X16077; Forward primer: 5′-CGGATAACCGTAGTAATTCTAG-3′, reverse primer: 5′-GTACTCATTCCAATTACCAGAC-3′). The Q-PCR was performed with Qbiogene Taq polymerase (MP Biomedicals, Illkirch, France) according to the manufacturer's protocol, and the amplification program was: 1 cycle at 95°C for 3 min, 40 cycles including one step at 95°C for 15 s, one at 60°C for 60 s and fluorescence reading. Lastly, a thermal decoupling was executed to obtain a melting curve to verify the specificity of the Q-PCR. To normalize our quantitative cycle (Cq) of the genes of interest (*BnSultr4;1* and *4;2*), a calculation was made as:

ΔCq = Quantitative cycle of the gene of interest (Cq) – (geometric average of quantitative cycle of internal control genes)
Then, the ΔΔCq method is applied as:
ΔΔCq = ΔCq treated sample – ΔCq control sample,
and the relative expression (RE) was calculated as:
RE = (1 + E)^−[ΔΔCq^] ≈ 2^−[ΔΔCq^]
where E = Q-PCR efficiency.

### Statistics

All data were performed with MINITAB13 on Windows® (Minitab Inc., State College, PA, USA). The normality of the data was studied with the Ryan–Joiner test at 95%. Analysis of variance (ANOVA) and the Tukey test were used to compare the means. When the normality law of data was not respected, the non-parametric test of Kruskal–Wallis was carried out. Statistical significance was postulated at *P* < 0.05. Four biological repetitions were used (*n* = 4) and all the data presented here are expressed as the mean value ± standard error (SE).

## Results

### Impact of S limitation occurring at the bolting stage (LS32 plants)

#### Growth, S amount, seed yield and seed quality

**Figure 1:**
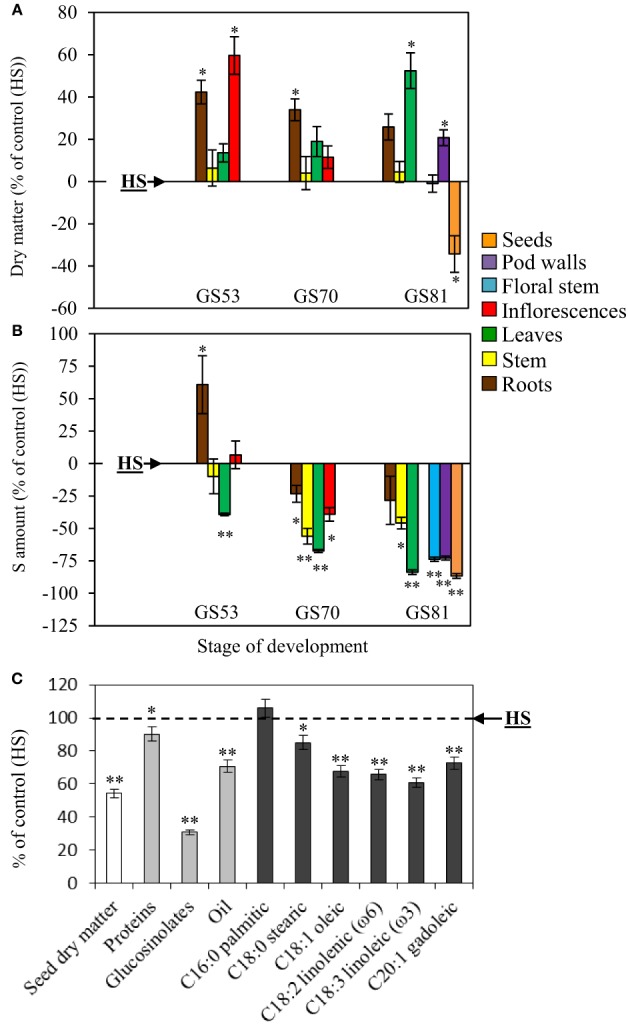
**Evolution of the dry matter (A), changes in the S amount (B) and seed yield and composition (C) of oilseed rape subjected to limitation of sulfate at the bolting stage**. S limitation (LS32 plants supplied with 8.7 μM sulfate) was compared to control plants (HS plants supplied with 508.7 μM sulfate). **(A)** Variations in dry matter of LS32 plants (as % of HS plants) between GS53 (early flowering), GS70 (start of pod filling) and GS81 (seed coloring). **(B)** Variations in the S amount in different organs of LS32 plants (as % of HS plants) between GS53 and GS81. A positive or a negative value indicates that the dry matter or the S amount is increased or reduced compared with HS plants, respectively. **(C)** Seed yield and composition of seeds (proteins, glucosinolates, oil, fatty acids) in LS32 plants (as % of HS plants) at mature seed stage (GS99). Significant differences between treatments are indicated with asterisks (*n* = 4; ^*^P < 0.05; ^**^P < 0.01).

When S limitation occurred at the bolting stage (LS32 plants), the dry matter of roots and inflorescences was significantly higher than in control (HS plants) with an increase of +42.3% (roots) and +59.7% (inflorescences) (Figure 1A). At the beginning of pod development (GS70), root dry matter was still significantly higher than in HS plants (+34%). At GS81 (seed coloring), an increase in dry matter was observed for leaves (+52.4%) and pod walls (+20.7%) in LS32 plants compared to control. As expected, a decrease in seed dry matter was observed at GS81 in response to S restriction (−34.3% compared to control HS plants; Figure 1A).

At the early flowering stage (GS53), the S amount increased only in roots (+60.8%) in response to sulfate limitation occurring at the bolting stage. As expected, at GS81, S limitation led to a decline in S amount in all aerial organs compared with the HS plants (Figure 1B). Thereafter, compared to HS plants, S amount decreased for all organs in later growing stages in LS32 plants.

At the mature seed stage (Figure 1C), the seed dry matter per plant was significantly reduced in LS32 plants (−54.3% compared to HS plants). Seed quality was also highly impacted by S limitation at the bolting stage. Protein (*p* < 0.05), glucosinolate (*p* < 0.01) and oil (*p* < 0.01) contents were lower in LS32 plants compared to HS plants. Oil composition was also significantly affected. Indeed, except for palmitic acid (C16:0) which did not differ from HS seeds, there was a decrease in all fatty acids, especially linoleic acid (C18:3 belonging to omega-3) and linolenic acid (C18:2 belonging to omega-6).

#### S management at the whole plant level: uptake, remobilization and loss by fallen leaves

**Figure 2:**
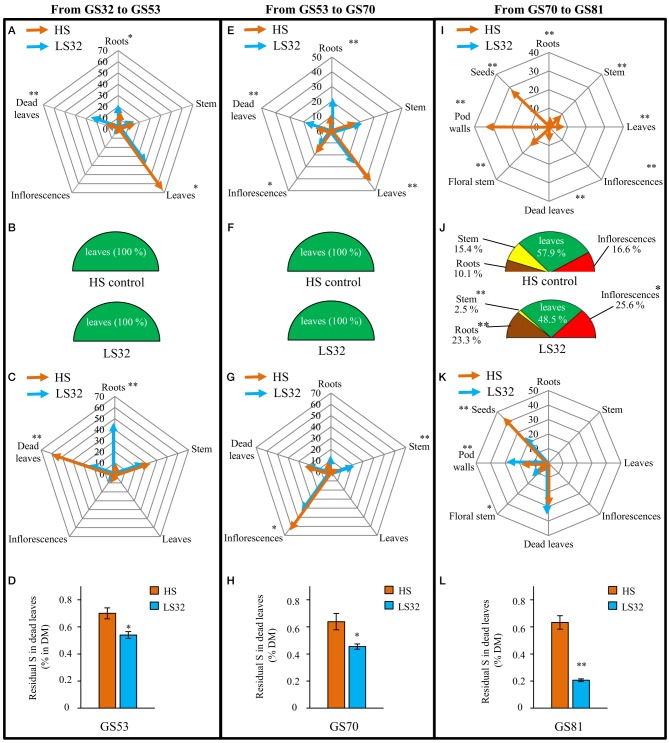
**Sulfur fluxes (A–C, E–G, I–K) and residual S in dead leaves (D,H,L) of oilseed rape subjected to limitation of sulfate at the bolting stage**. S limitation (LS32 plants supplied with 8.7 μM sulfate) was compared to control plants (HS plants supplied with 508.7 μM sulfate) between GS32 (bolting stage) and GS81 (seeds coloring). Flux of S taken up from the soil allocated to the sink organs are indicated as % of total S taken up between **(A)** GS32 and GS53 (early flowering), **(E)** GS53 and GS70 (start of pod filling) and **(I)** GS70 and GS81 (seeds coloring). The diagrams **(B,F,J)** represent the contribution of source organs to the S remobilization in HS and LS32 plants between GS32 and GS53, GS53 and GS70, and GS70 and GS81, respectively (data are expressed as % of total S remobilized). Flux of S remobilized from the source organs toward the sink organs are mentioned as % of total remobilized S between **(C)** GS32 and GS53, **(G)** GS53 and GS70, and **(K)** GS70 and GS81. Residual S in dead leaves (as % of dry matter) of HS and LS32 plants are indicated at **(D)** GS53, **(H)** GS70 and **(L)** GS81. Significant differences between treatments are indicated with asterisks (*n* = 4; ^*^P < 0.05; ^**^P < 0.01).

The ^34^S labeling pulse-chase method used in this experiment was performed to determine the exogenous S flux (from S uptake) and endogenous S flux (from remobilization of S reserves) at different stages of development (Figure 2).

Between bolting (GS32) and the early flowering (GS53) stages (Figure 2A), the S taken up from the soil in control (HS) plants was mainly allocated to leaves (64.1% of total S uptake) with nearly equal amounts going to roots and stems (11.3 and 12.3%, respectively). A significant part of this S is lost through dead leaves (11.3%). Despite the low level of S fertilization, a low but significant S uptake was observed in LS32 plants and this S taken up was mainly allocated to leaves, but in a smaller proportion (40.2%) than in HS plants (Figure 2A). The determination of S remobilization fluxes (Figures 2B,C) indicated that leaves were the only source organs in both sulfate treatments. The main sink for S remobilized from source leaves was the stems in HS plants (31.3%) and roots in LS32 plants (20%) (Figure 2C). This large redistribution to roots in LS32 plants can be explained by the increase in root dry matter (Figure 1A) and can explain the increase in total S in this organ (Figure 1B). Surprisingly, more than a half of the remobilized S was lost through dead leaves in HS plants (58.7% of total remobilized S). By contrast, only 23.3% of remobilized S was lost due to leaf fall in LS32 plants (Figure 2C).

Between the early flowering stage (GS53) and the start of pod filling (GS70) (Figure 2E), leaves were the main sink organs for S taken up in HS plants (42.2%) and the remainder was allocated to inflorescences (20%), stem (17.3%) and roots (10.8%). The proportion of S lost by dead leaves was the same as in previous growing stages (about 10%). For LS32 plants, exogenous S was still taken up between GS53 and GS70 and was equally distributed between leaves (25%), roots (22%) and stem (21.2%). As before, the increase in root sink strength in response to LS32 treatment was probably due to the increase in dry matter (Figure 1A). The S remobilization fluxes (Figures 2F,G) revealed that (i) leaves were the only source organs (Figure 2F) and (ii) the inflorescences were the main sink organs in both S treatments (60.8% for HS and 41.3% for LS32 plants; Figure 2G). S was highly remobilized to stems in response to LS32 treatment (22.7%) compared to HS plants (4.5%; Figure 2G). The same proportion of remobilized S was lost by dead leaves in both treatments (22.73 and 20% for HS and LS32 plants, respectively) (Figure 2G).

Unlike HS plants, there was no S uptake by LS32 plants between the onset of pod filling (GS70) and the seed coloring (GS81) stages (Figure 2I). In HS plants, the main sinks for S taken up were pod walls (33.3% of total S taken up) and seeds (28%). The S taken up that was lost by dead leaves at this time was lower than in previous stages of development but was still significant (7.5%; Figure 2I). The leaves were the main source organs for S remobilization (57.8 and 48.5% for HS and LS32 plants, respectively; Figure 2I). The contribution of roots and inflorescences as source organs for remobilized S in LS32 plants was stronger in response to S limitation (23.3% for roots; 25.6% for inflorescences) than in HS plants (10% for roots; 16.6% for inflorescences) (Figure 2J). In response to LS32 treatment a larger part of the remobilized S was distributed to pod walls (30.3% compared to 17.4% for HS plants) to the detriment of seeds (21.5% compared to 43.2% for HS plants; Figure 2K). As previously observed between GS53 and GS70, the proportion of S lost by dead leaves was the same in both S treatments between GS70 and GS81 (Figure 2K).

The residual S percentage in fallen leaves did not change during the whole experiment in HS plants (0.67 ± 0.03% in average; Figures 2D,H,L). On the contrary, in LS32 plants, a decrease in residual S in dead leaves was observed from GS53 and it is lower than in HS plants at every growing stage (0.54, 0.45 and 0.20% at GS53, GS70 and GS81, respectively; Figures 2D,H,L).

#### Involvement of sulfate and vacuolar sulfate transporters in S remobilization of source leaves

**Figure 3:**
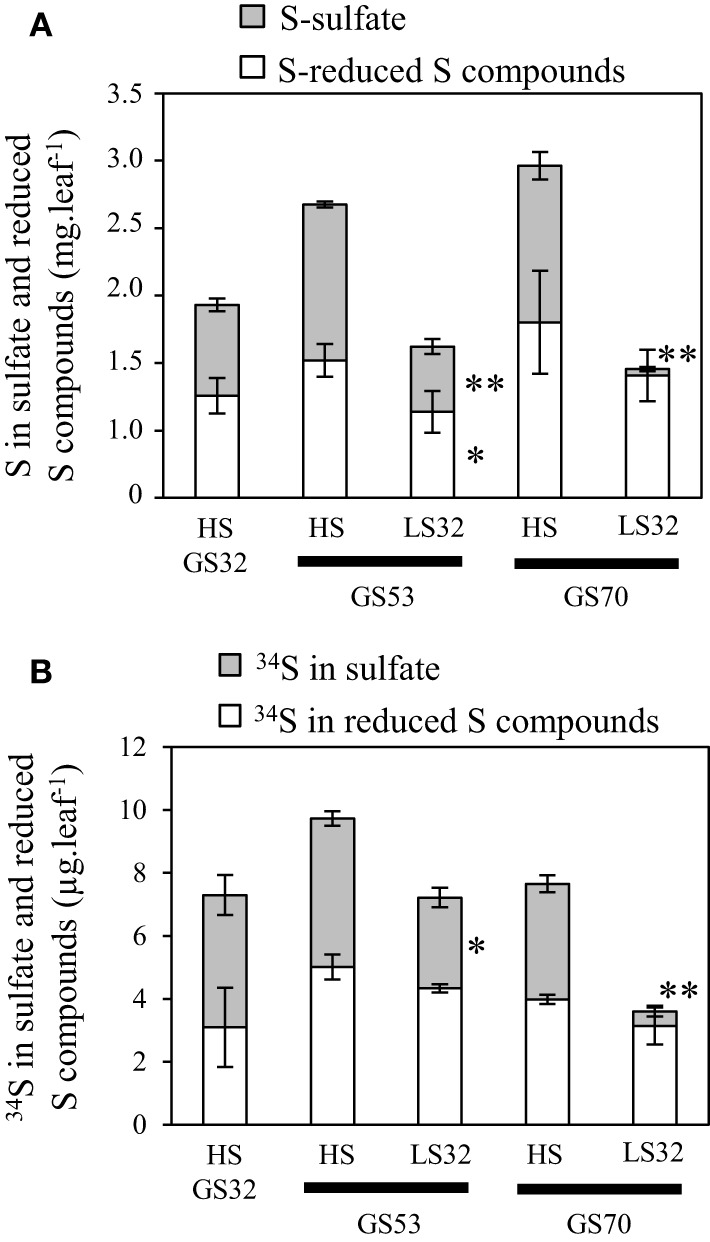
**Changes in S (A) and ^34^S (B) amount determined in the sulfate and reduced S-compounds fractions in source leaves of oilseed rape subjected to limitation of sulfate at the bolting stage**. S limitation (LS32 plants supplied with 8.7 μM sulfate) was compared to control plants (HS plants supplied with 508.7 μM sulfate) between GS32 (bolting stage), GS53 (early flowering) and GS70 (start of pod filling). Significant differences between treatments are indicated with asterisks (*n* = 4; ^*^P < 0.05; ^**^P < 0.01).

**Figure 4:**
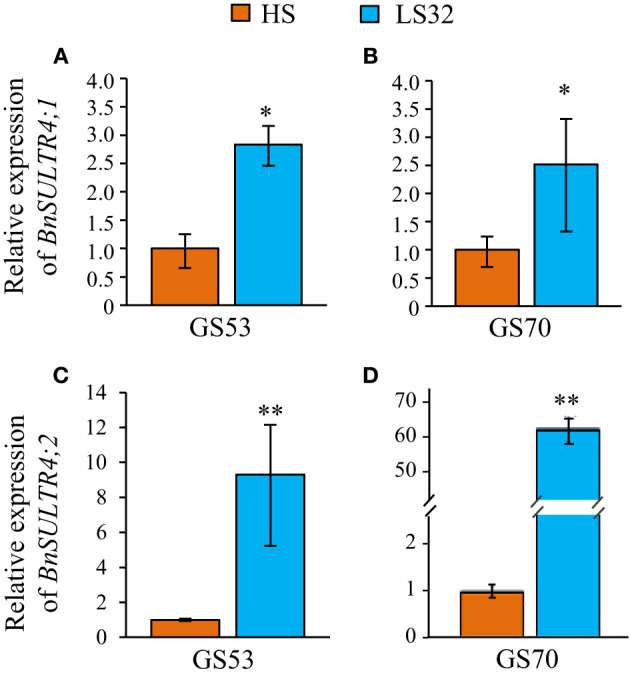
**Relative expression of *BnSultr4;1* (A,B) and *BnSultr4;2* (C,D) genes observed at GS53 (early flowering) and GS70 (start of pod filling) in source leaves of oilseed rape subjected to limitation of sulfate at the bolting stage**. S limitation (LS32 plants supplied with 8.7 μM sulfate) was compared to control plants (HS plants supplied with 508.7 μM sulfate). For each stage of development, the value 1 is attributed for the level of transcripts observed in source leaves of HS plants. Significant differences between treatments are indicated with asterisks (*n* = 4; ^*^P < 0.05; ^**^P < 0.01).

Analysis of S fluxes has revealed that leaf S remobilization was improved in response to an S deficiency. Therefore, the dynamics of sulfate and S-reduced compounds (including S-amino acids, glutathione, proteins and other S-organic compounds) in source leaves were investigated in relation to (i) flux of leaf S and ^34^S remobilization and (ii) transcript levels of the *BnSULTR4;1* and *4;2* transporters involved in vacuolar efflux of sulfate. In response to S restriction occurring at the bolting stage, the decrease in S in source leaves was mainly related to a strong decline of sulfate while S-reduced compounds slightly decreased at the early flowering stage (GS53) or remained constant at the onset of pod filling (GS70; Figure 3A). Moreover, a decrease of ^34^S in the sulfate fraction was observed at GS53 and GS70 for LS32 plants, while the ^34^S in S-reduced compounds remained constant at these growth stages (Figure 3B). In parallel to this decrease in sulfate, an induction of *BnSULTR4;1* transcripts was observed in source leaves of LS32 plants (3-fold and 2.5-fold higher than in HS plants at GS53 and GS70, respectively; Figures 4A,B). Compared to HS plants, the transcript level of *BnSULTR4;2* in source leaves of LS32 plants was highly up-regulated (9 and 60-fold higher at GS53 and GS70, respectively; Figures 4C,D).

### Impact of S limitation occurring at the early flowering stage (LS53 plants)

#### Growth, S amount, seed yield and seed quality

**Figure 5:**
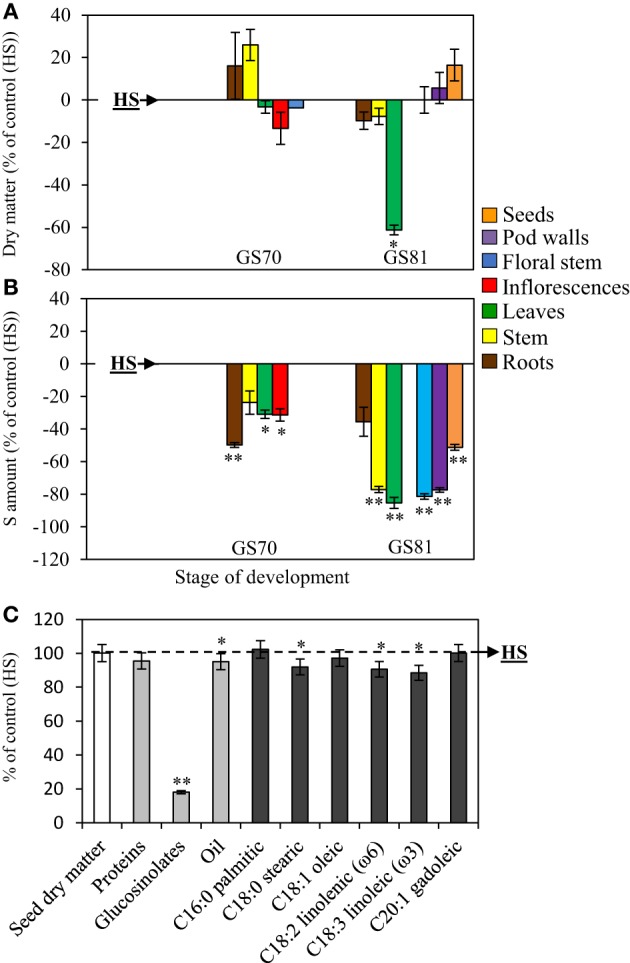
**Evolution of the dry matter (A), changes in the S amount (B) and seed yield and composition (C) of oilseed rape subjected to limitation of sulfate at the early flowering stage**. S limitation (LS53 plants supplied with 8.7 μM sulfate) was compared to control plants (HS plants supplied with 508.7 μM sulfate). **(A)** Variations in dry matter of LS53 plants (as % of HS plants) between GS70 (start of pod filling) and GS81 (seed coloring). **(B)** Variations in the S amount in different organs of LS53 plants (as % of HS plants) between GS70 and GS81. A positive or a negative value indicates that the dry matter or the S amount is increased or reduced compared with HS plants, respectively. **(C)** Seed yield and composition of seeds (proteins, glucosinolates, oil, fatty acids) in LS53 plants (as % of HS plants) at mature seed stage (GS99). Significant differences between treatments are indicated with asterisks (*n* = 4; ^*^P < 0.05; ^**^P < 0.01).

Compared to control (HS plants), a limitation of sulfate occurring at the early flowering stage (LS53 plants) had no effect on dry matter of all organs except for dry matter of leaves at GS81 (-61.3% compared to HS, Figure 5A). The S amount of LS53 plants is lower than HS plants in all organs at every growing stage, except for stem at GS70 and roots at GS81, for which there were no significant differences compared to HS plants (Figure 5B). The seed dry matter per plant (Figure 5C) was not affected by S limitation. In addition, protein content was not altered, in contrast to the glucosinolate content, which decreased by 82% compared to HS plants (Figure 5C). Oil content and oil quality were also slightly reduced in LS plants (*P* < 0.05) due to the decrease in linoleic and linolenic acid content (−9.4 and −11.5%, respectively, compared to HS plants) (Figure 5C).

#### S management at the whole plant level: uptake, remobilization and loss by fallen leaves

**Figure 6:**
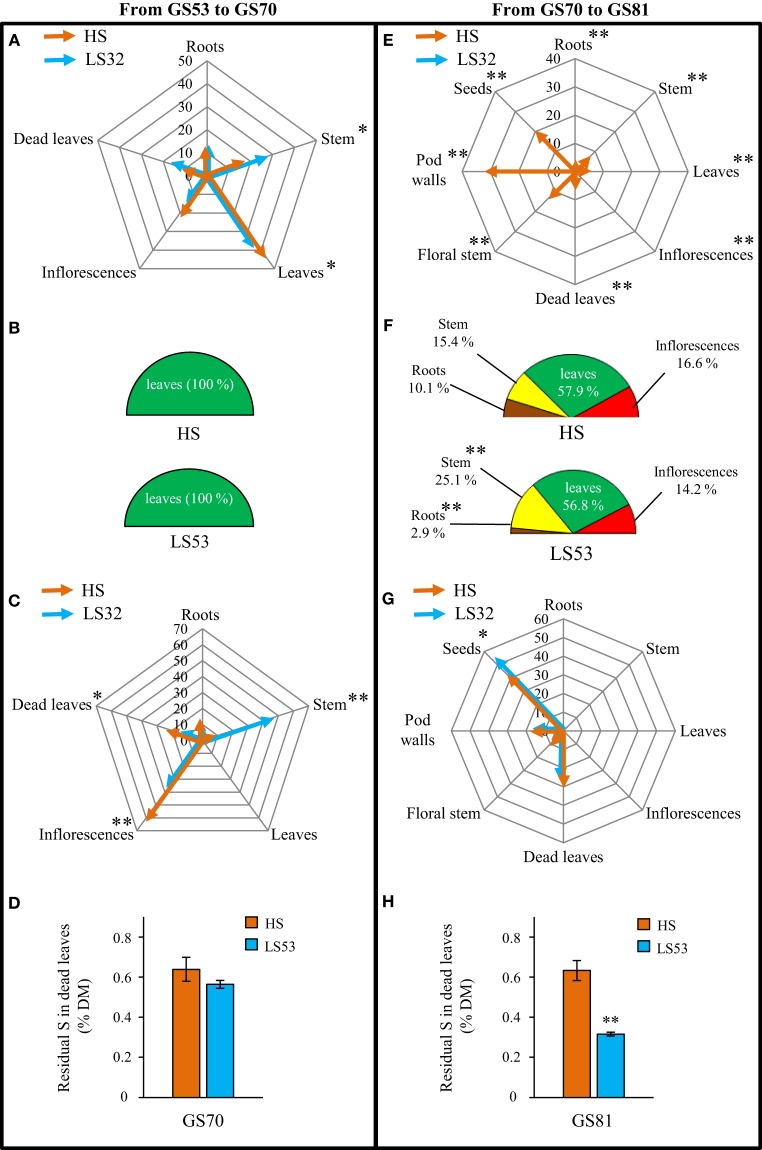
**Sulfur fluxes (A–C, E–G) and residual S in dead leaves (D,H) of oilseed rape subjected to limitation of sulfate at the early flowering stage**. S limitation (LS53 plants supplied with 8.7 μM sulfate) was compared to control plants (HS plants supplied with 508.7 μM sulfate) between GS53 (early flowering stage) and GS81 (seeds coloring). Flux of S taken up from the soil allocated to the sink organs are indicated as % of total S taken up between **(A)** GS53 and GS70 (start of pod filling) and **(E)** GS70 and GS81 (seeds coloring). The diagrams **(B,F)** represent the contribution of source organs to the S remobilization in HS and LS53 plants between GS53 and GS70, and GS70 and GS81, respectively (data are expressed as % of total S remobilized). Flux of S remobilized from the source organs toward the sink organs are mentioned as % of total remobilized S between **(C)** GS53 and GS70, and **(G)** GS70 and GS81. Residual S in dead leaves (as % of dry matter) of HS and LS53 plants are indicated at **(D)** GS70 and **(H)** GS81. Significant differences between treatments are indicated with asterisks (*n* = 4; ^*^: *P* < 0.05; ^**^: *P* < 0.01).

Between early flowering (GS53) and the onset of pod filling (GS70), the main sink organs for S taken up in LS53 plants were leaves (34% of total S from the soil; Figure 6A). The main difference between HS and LS53 plants was the sink strength of stems. Indeed, the allocation of S taken up into the stem increased for LS53 plants (25.3% compared to 17.3% for HS plants). In both S treatments, leaves were the main source organs for remobilized S between GS53 and GS70 (Figure 6B). A larger proportion of the remobilized S was redistributed to stem in LS53 plants (47.4%) compared to HS plants (4.5%) (Figure 6C). These results explain the lack of difference between the total S amount in stems of HS and LS53 plants at GS70. Moreover, in response to LS53 treatment, stem dry matter slightly increased, but it was not significantly different to HS plants. Therefore, the increase in S taken up and the redistribution of remobilized S to stems cannot be explained by an increase of growth. As expected for LS53 plants, a smaller proportion of remobilized S was lost through dead leaves (13.55% for LS53 plants vs. 22.73% for HS plants, Figure 6C).

Between the beginning of pod filling (GS70) and the seed coloring stage (GS81), there was no S uptake for LS53 plants while the S taken up in HS plants was essentially allocated to pods and seeds (Figure 6E). Leaves were the major source organs for remobilized S in LS53 and HS plants (Figure 6F). In addition, stems represented a more important source for S remobilization in LS53 plants (25% of total remobilized S) than HS plants (15.4%; Figure 6F). The main sink organs for remobilized S were seeds for both S treatments but especially for LS53 plants with 53.7% of remobilized S redistributed to seeds vs. 43.2% in HS plants (Figure 6G). This larger S allocation and remobilization toward seeds could explain why LS53 plants had a similar seed yield to HS plants despite S restriction. At the onset of pod filling (GS70) the residual S percentage in dead leaves of LS53 plants was slightly decreased compared to HS plants, but not significantly (0.64 and 0.56%, respectively; Figure 6D). In contrast, at the seed coloring stage (GS81), a significant decrease was observed (0.64% and 0.31% for HS and LS53 plants, respectively; Figure 6H).

#### Involvement of sulfate and vacuolar sulfate transporters in S remobilization of source leaves

**Figure 7:**
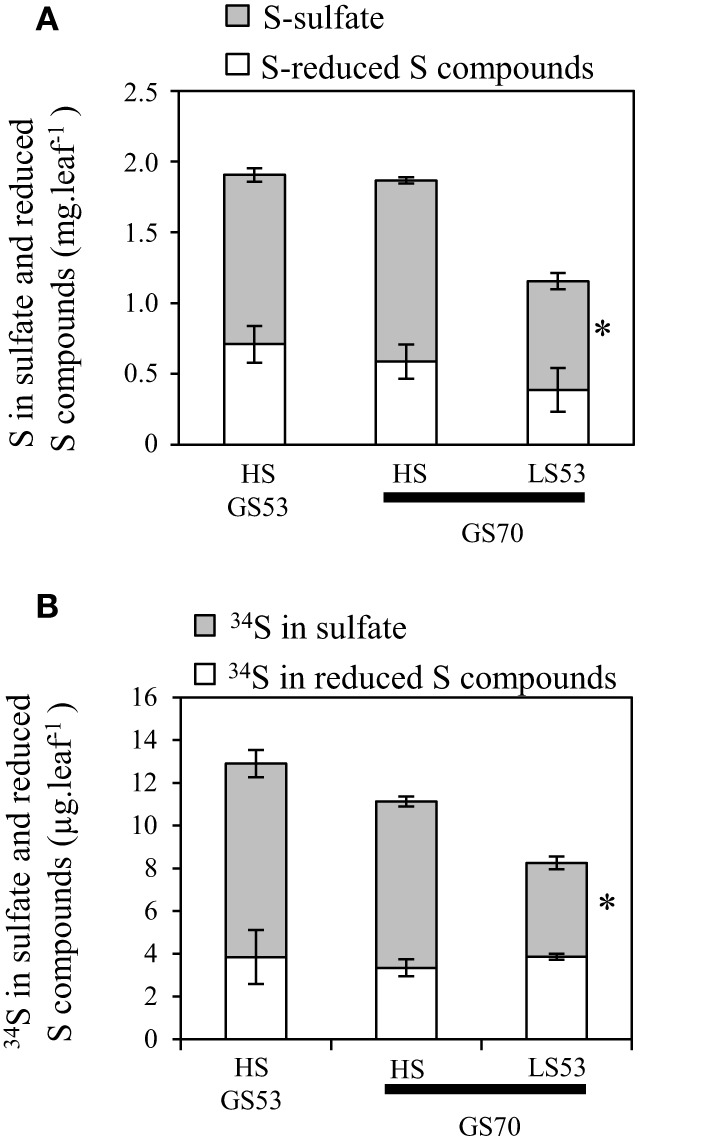
**Changes in S (A) and ^34^S (B) amounts determined in the sulfate and reduced S-compounds fractions in source leaves of oilseed rape subjected to limitation of sulfate at the early flowering stage**. S limitation (LS53 plants supplied with 8.7 μM sulfate) was compared to control plants (HS plants supplied with 508.7 μM sulfate) between GS53 (early flowering) and GS70 (start of pod filling). Significant differences between treatments are indicated with asterisks (*n* = 4; ^*^P < 0.05; ^**^P < 0.01).

**Figure 8:**
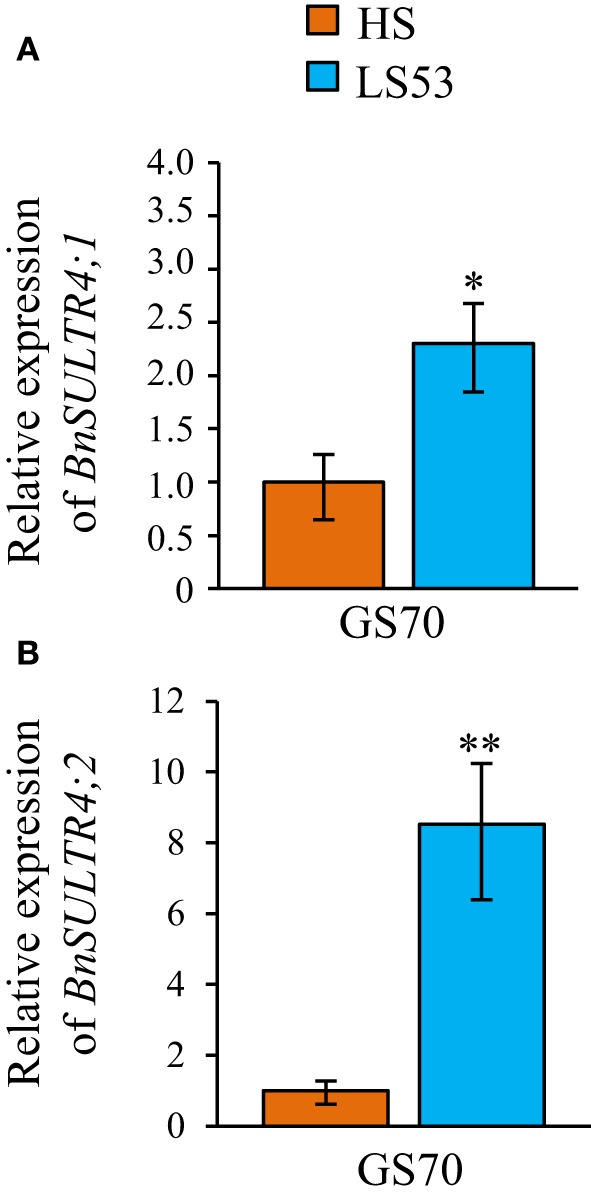
**Relative expression of *BnSultr4;1* (A) and *BnSultr4;2* (B) genes observed at GS70 (start of pod filling) in source leaves of oilseed rape subjected to limitation of sulfate at the early flowering stage**. S limitation (LS53 plants supplied with 8.7 μM sulfate) was compared to control plants (HS plants supplied with 508.7 μM sulfate). For each stage of development, the value 1 is attributed for the level of transcripts observed in source leaves of HS plants. Significant differences between treatments are indicated with asterisks (*n* = 4; ^*^P < 0.05; ^**^P < 0.01).

At GS70, a decrease in the amount of sulfate and ^34^S-sulfate was noted in source leaves of LS53 plants while the S and ^34^S in S-reduced compounds remained constant (Figures 7A,B). This decrease in sulfate was concomitant with up-regulation of *BnSULTR4;1* (2.3-fold) and *BnSULTR4;2* (8-fold) expression in source leaves of LS53 plants compared to HS plants (Figures 8A,B).

## Discussion

### S supply before the flowering stage is crucial for seed yield and seed quality in winter oilseed rape

The link between S availability, seed yield and seed quality of cereals and oilseed crops has been described previously (Janzen and Bettany, 1984; Zhao et al., 1997; Hawkesford, 2000; Scherer, 2001; Ahmad et al., 2007). Nevertheless, the stages of development that are the more sensitive to sulfate limitation and the source/sink relationships for S, and more particularly the contribution of S from absorption or remobilization in the reallocation of S to seeds, remain largely unclear in winter oilseed rape. The aims of this study were to determine how oilseed rape was able to manage its S reserves in order to maintain the seed yield and seed quality when sulfate limitation (LS) occurred at bolting stage (LS32 treatment) or early flowering stage (LS53 treatment). A sulfate limitation applied at the bolting stage (LS32 plants) affects seed yield, and seed quality with lower proteins and oil content (Figure 2C). Moreover, oil quality was affected leading to a decline in many fatty acids especially linoleic and linolenic acids which are particularly required in human nutrition. When S limitation occurred at the early flowering stage (LS53 plants), seed yield was the same as in control plants, but linoleic and linolenic acid contents were affected too. On the contrary, as shown by Dubousset et al. (2010), S restriction applied at the onset of pod filling did not affect seed yield and oil quality. The same results on grain quality were obtained in wheat by Anderson and Fitzgerald (2001) when S restriction occurred after anthesis. These results show that the S supply before the anthesis is crucial to sustain S demand for seed growth and to guarantee an optimal seed yield and seed quality. Moreover, the glucosinolate content in mature seeds declined when S limitation was applied at bolting or early flowering stages (Figures 1C, 5C). These secondary metabolites may represent a problem for meal production because a high level of some glucosinolates can affect palatability (Robbelen and Thies, 1980). On the other hand, glucosinolates may serve, *via* the myrosinase activity, as precursor of isothiocyanates, thiocyanates, nitriles, or epithionitriles that are important in defense mechanisms against pathogens (Halkier and Gershenzon, 2006) as demonstrated in leaves of oilseed rape (Dubuis et al., 2005). Interestingly, D'Hooghe et al. (2014) have reported that the mature seeds of LS53 and LS32 plants were characterized by a low myrosinase abundance. Even if the low level of glucosinolates and myrosinase observed in mature seeds in response to S limitation applied before anthesis may improve meal palatability, these data suggest that these S-limited seeds may be more susceptible to pathogens attacks.

### S uptake and S remobilization efficiency are differently impacted by the sulfate limitation occurring at the bolting or the early flowering stages

In response to S limitation applied at the bolting stage (LS32 plants), seed yield and quality were drastically affected meaning that the S management by the LS32 plants was not efficient to compensate the low sulfate availability. However, our study revealed that LS32 plants significantly increased their root dry matter (+42.3% compared to HS plants, Figure 1A). It was also well established that the two high-affinity sulfate transporters (*Sultr1;1* and *Sultr1;2*) were involved in the major part of sulfate uptake (Davidian and Kopriva, 2010), and that gene expression of *BnSultr1;1* and *BnSultr1;2* was up-regulated in roots of oilseed rape subjected to S restriction (Buchner et al., 2004a,b; Parmar et al., 2007; Dubousset et al., 2009). In our experiment, the enhancement of root dry matter was concomitant with the increase in root sink strength for S (Figures 2A,C) indicating an increase in the sulfate uptake capacities as previously reported in oilseed rape at the vegetative stage (Abdallah et al., 2010) or in *Arabidopsis* (Hoefgen and Nikiforova, 2008). In our experimental conditions (culture in pot containing perlite and vermiculite with a very low sulfate availability), the increase of sulfate absorption capacities observed in LS32 plants did not lead to sustain the S demand for growth of reproductive organs. Although the remobilization of S from leaves was strongly induced in LS32 plants to sustain the root growth between the bolting and early flowering stages (Figure 2C), this was not sufficient to satisfy the S requirements for seed development and filling. However, under field conditions, the improvement of S absorption efficiency in response to limitation of S fertilizers *via* an increase of root proliferation and the induction of sulfate transporters in roots may help to explore a more important volume of soil and consequently maximize uptake of mineral S (Hawkesford, 2000). These physiological traits (increase of S remobilization from leaves to sustain the root proliferation and the S uptake efficiency) may limit the negative impacts on the seed yield and quality in case of low availability of sulfate occurring before the bolting stage.

When S limitation occurred at the early stages of flowering (LS53 plants), the S use efficiency of oilseed rape was improved to maintain seed yield and quality (Figure 5C). To understand the compensation mechanisms that occur in LS53 plants, the S fluxes were investigated and demonstrated that no matter what growing stages are considered, leaves are the most important source organs for S. Except at the onset of pod development (GS70), where the S amount was identical between LS53 and HS plants (Figure 6D), the residual S amount in leaves was lower in LS53 plants than in HS plants, which implies a better foliar S remobilization in S-limited plants (Figure 6H). Between the early flowering and the beginning of pod development, the remobilized S from leaves was mainly redistributed toward the stem in LS53 plants (Figure 6C). Between the onset of pod development and the seed coloring stages, the remobilization of S toward seeds was greater in LS53 plants than HS plants (Figure 6G). In parallel, the contribution of source leaves to S remobilisation was similar between LS53 and HS plants while the stem represented a major source organ for S remobilization in LS53 plants (25% of the total remobilized S vs. 15.4% for HS plants; Figure 6F). These results support the hypothesis that, in response of S restriction occurring at the flowering stages, the stem is a transient storage organ for S and may have a pivotal function in the case of asynchronism between S remobilization from source leaves and S use by reproductive organs.

### Sulfate is the main S compound remobilized in source leaves

Once taken up from the soil, S in the form of sulfate is assimilated or transiently stored in vacuoles of roots or leaves (Davidian and Kopriva, 2010). Sulfate is the main storage form of S in Brassicacea. Indeed, Blake-Kalff et al. (1998) showed that in leaves of oilseed rape well supplied with S, sulfate can represent 70–90% of the total S amount. Several studies have suggested that vacuolar sulfate is a major form for S remobilization in response to S restriction at the vegetative stages in *Arabidopsis* or oilseed rape (Hawkesford, 2000; Kataoka et al., 2004; Parmar et al., 2007; Dubousset et al., 2009).

For LS32 and LS53 plants, a diminution in the total amount of S was observed in parallel to a decrease in the amount of sulfate while the amount of S-reduced compounds remained constant or slightly decreased (Figures 3A, 7A). Moreover, the ^34^S-sulfate decreases but not the ^34^S in S-reduced compounds. These results indicate that sulfate is the main S compound remobilized in leaves of plants subjected to an S restriction that is applied at the bolting and early flowering stages. In addition, an up-regulation of *BnSULTR4;1* and *BnSULTR4;2* gene expression was observed alongside the decrease in sulfate. After the bolting stage, sulfate destined for remobilization is probably derived from sulfate previously stored in vacuoles of source leaves. The increase in gene expression of these two SULTR4-type transporters has been shown at the vegetative stage in old and mature leaves (Dubousset et al., 2009) and in roots (Parmar et al., 2007) of oilseed rape. Moreover, Parmar et al. (2007) have reported that the transcript abundance of *BnSULTR4;2* is inversely correlated with sulfate concentration trends in tissues of *Brassica napus* at the vegetative stage. Although other transporters are supposed to play a significant role in sulfate remobilization, including transport from cytoplasm to phloem such as the transporters of groups 1 or 2, our results highlight the contribution of tonoplastic SULTR4-type transporters in the efflux of sulfate from the vacuole of source leaves in response to S restriction occurring at the reproductive stages (Buchner et al., 2004b).

In conclusion, our findings clearly indicate that leaves are the most important source organs for S during reproductive stages of oilseed rape. By combining ^34^S-labeling with biochemical fractionation in order to separate sulfate from other S-compounds, the present study shows that sulfate is the main form of S remobilized in leaves at reproductive stages and that tonoplastic SULTR4-type transporters are particularly involved in the sulfate remobilisation in case of low S availability. Nevertheless, further experiments need to be performed to determine if sulfate itself is transported by the phloem to young pods and seeds or if it is reduced to other transportable S compounds. Our investigations on S fluxes at whole plant level also reveal that (i) the induction of root proliferation may help to maximize the sulfate uptake capacities especially when S limitation appeared at the bolting stage and (ii) the stem may serve as a transient storage organ for S in response to S limitation occurred at the early flowering stage. Consequently, these physiological traits (sulfate remobilization, root proliferation and/or transient S storage in stem) could be used in breeding programs to select oilseed rape genotypes with high S use efficiency or able to limit the impact of mineral S limitation on seed yield and quality.

## Author contributions

All authors contributed to the experimental design, to plant growth and tissue sampling and have been involved in revising the article critically for important intellectual content. Alexandra Girondé, Lucie Dubousset, Jacques Trouverie and Jean-Christophe Avice carried out the S flux calculations. Alexandra Girondé and Jean-Christophe Avice performed the biochemical fractionation. Alexandra Girondé and Philippe Etienne performed the molecular analyses for determination of gene expression of SULTR4-type transporters. Alexandra Girondé and Lucie Dubousset made other measurements and analyses, including statistical analyses, interpretation of data and drafting the article.

### Conflict of interest statement

The Associate Editor Stanislav Kopriva declares that, despite having collaborated with authors Lucie Dubousset, Jacques Trouverie and Jean-Christophe Avice, the review process was handled objectively and no conflict of interest exists. The authors declare that the research was conducted in the absence of any commercial or financial relationships that could be construed as a potential conflict of interest.

## Abbreviations

DM: Dry Matter
FAME: fatty acid methyl ester
GC: gas chromatography
GS: growth stage
HS: High S
LS: Low S
NIRS: Near Infrared Spectroscopy
S: Sulfur
V-CDT: Vienna-Canyon Diablo Troilite.
