# Supplementary material for: The impact of sulfate restriction on seed yield and quality of winter oilseed rape depends on the ability to remobilize sulfate from vegetative tissues to reproductive organs
Source: Front Plant Sci. 2014 Dec 17;5:695. doi: 10.3389/fpls.2014.00695 (PMC4269117; doi:10.3389/fpls.2014.00695)
Supplement: Supplementary file 1 [file Presentation1.PPT]

## Slide 1
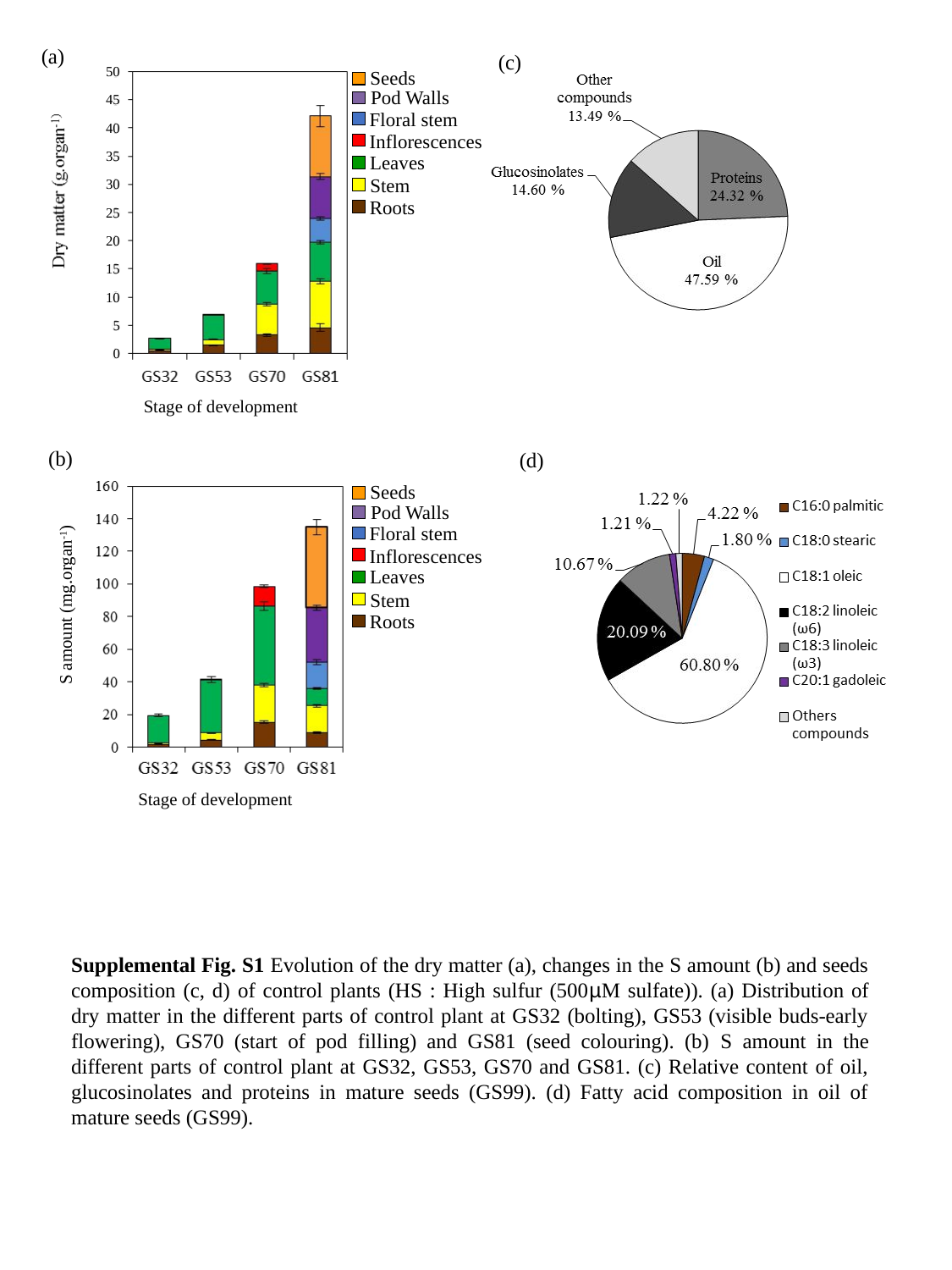

(a)
Stage of development
Seeds
Floral stem
Inflorescences
Leaves
Stem
Roots
Pod Walls
50
45
40
35
30
25
20
15
10
 5
 0
(c)
(d)
(b)
 S amount (mg.organ-1)
Stage of development
Seeds
Floral stem
Inflorescences
Leaves
Stem
Roots
Pod Walls
Supplemental Fig. S1 Evolution of the dry matter (a), changes in the S amount (b) and seeds composition (c, d) of control plants (HS : High sulfur (500µM sulfate)). (a) Distribution of dry matter in the different parts of control plant at GS32 (bolting), GS53 (visible buds-early flowering), GS70 (start of pod filling) and GS81 (seed colouring). (b) S amount in the different parts of control plant at GS32, GS53, GS70 and GS81. (c) Relative content of oil, glucosinolates and proteins in mature seeds (GS99). (d) Fatty acid composition in oil of mature seeds (GS99).
